# Tumor‐educated platelets in cancer diagnostics and prognostics: A critical appraisal and roadmap for clinical translation

**DOI:** 10.1002/ijc.70423

**Published:** 2026-03-07

**Authors:** Whi‐An Kwon, Min‐Kyung Lee, Eunyong Ahn, Heeyeon Kim, Yong Sang Song, Taejin Ahn

**Affiliations:** ^1^ Department of Urology Hanyang University College of Medicine, Myongji Hospital Goyang Republic of Korea; ^2^ Research Institute of Precision Medicine and Geroscience Myongji Medical Foundation Goyang Republic of Korea; ^3^ Department of Internal Medicine Hanyang University College of Medicine, Myongji Hospital Goyang Republic of Korea; ^4^ Research Group, Foretell My Health Pohang Republic of Korea; ^5^ Department of Obstetrics and Gynecology Hanyang University College of Medicine, Myongji Hospital Goyang Republic of Korea; ^6^ Department of Life Science, Handong Global University Pohang Republic of Korea

**Keywords:** cancer diagnosis, clinical validation, early cancer detection, liquid biopsy, pan‐cancer screening, platelet RNA, tumor‐educated platelet (TEP)

## Abstract

Tumor‐educated platelets (TEPs) are emerging as a compelling frontier in liquid biopsy, functioning as dynamic, systemic sensors that sequester and process tumor‐derived biomolecules. This interaction imprints an integrated molecular signature of malignancy—spanning the transcriptome, proteome, lipidome, and crucially, the captured genome—within the circulating platelet population. Recent mechanistic evidence suggests that platelets can sequester extracellular DNA, including circulating tumor DNA (ctDNA), and may thereby act as a relatively protected, time‐integrated reservoir that extends the biological persistence of tumor‐derived fragments beyond the short plasma half‐life (tens of minutes to a few hours). This emerging platelet‐sequestered DNA (pDNA) signal complements more established platelet transcriptomic data; to date, most TEP‐based molecular profiling has relied on RNA and has shown promising performance in identifying diverse solid malignancies, including premalignant lesions, and in some settings facilitating localization of the tissue of origin. However, the translation of these findings into clinical‐grade assays is obstructed by substantial hurdles. Pre‐analytical inconsistencies (e.g., leukocyte/erythrocyte contamination, isolation protocol choice) introduce technical noise and pervasive batch effects that compromise reproducibility. These vulnerabilities have been underscored by failed external validations and poor generalizability of discovery‐phase classifiers in independent cohorts and prospective settings. This review critically dissects the factors influencing TEP diagnostic performance and outlines a concrete roadmap for clinical translation. We argue that rigorous standardization—universal standard operating procedures, harmonized pre‐analytics, and quantitative quality‐control metrics—is the sine qua non for implementation, to be followed by large‐scale, prospective validation trials designed to demonstrate clinical utility and by multi‐omics integration to maximize diagnostic yield.

## Graphical abstract

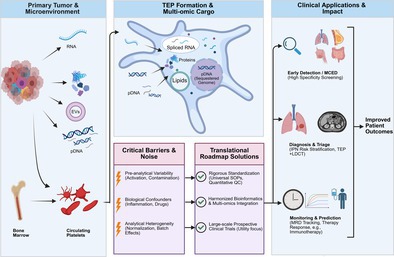

**Abbreviations**
*AR‐V7*
: Androgen Receptor splice variant 7
AUC: area under the receiver operating characteristic curve
BLOODPAC: blood profiling atlas in cancer
Ca: cancer
CD62P: P‐selectin (platelet activation marker)
CDx: companion diagnostic
cfDNA: cell‐free DNA
CHIP: clonal hematopoiesis of indeterminate potential
CNV: copy number variation
CTAP3: connective tissue‐activating peptide 3
CTC: circulating tumor cell
ctDNA: circulating tumor DNA
CUP: cancer of unknown primary
ddPCR: droplet digital polymerase chain reaction
EDTA: ethylenediaminetetraacetic acid
*EGFRvIII*
: epidermal growth factor receptor variant III
ELBS: European Liquid Biopsy Society
EMA: European Medicines Agency
EV: extracellular vesicle
FDA: US Food and Drug Administration
GBM: glioblastoma multiforme
GI: gastrointestinal
IFN‐γ: interferon‐gamma
IPN: indeterminate pulmonary nodule
ISTH: International Society on Thrombosis and Hemostasis
*KLK3*
: Kallikrein‐related peptidase 3
LDCT: low‐dose computed tomography
LDT: laboratory developed test
lncRNA: long non‐coding RNA
MCED: multi‐cancer early detection
MRD: minimal residual disease
MTDE: minimum technical data element
N/A: not applicable
NGS: next‐generation sequencing
NPC: nasopharyngeal carcinoma
NPV: negative predictive value
NSCLC: non–small cell lung cancer
PAC‐1: monoclonal antibody recognizing activated integrin αIIbβ₃
*PCA3*
: *Prostate Cancer Antigen 3*
PD‐1: programmed cell death protein 1
PDGF: platelet‐derived growth factor
PD‐L1: programmed death‐ligand 1
pDNA: platelet‐sequestered DNA
PF4: platelet factor 4
PLS‐DA: partial least squares–discriminant analysis
pPD‐L1: platelet programmed death‐ligand 1
PPV: positive predictive value
PRP: platelet‐rich plasma
QC: quality control
qPCR: quantitative polymerase chain reaction
RCC: renal cell carcinoma
RCT: randomized controlled trial
RNA‐seq: RNA sequencing
ROC: receiver operating characteristic
SOP: standard operating procedure
SROC: summary receiver operating characteristic
SSC: Scientific and Standardization Committee
TEP: tumor‐educated platelet
TSP‐1: thrombospondin‐1
VEGF: vascular endothelial growth factor

## 1 INTRODUCTION: TUMOR‐EDUCATED PLATELETS AT THE CROSSROADS OF PROMISE AND CLINICAL REALITY

Tumor‐educated platelets (TEPs) have emerged as a key frontier in liquid biopsy, acting as dynamic biosensors of malignancy. Discovery and multi‐cancer studies indicate that platelet RNA profiles can detect cancer with high specificity and, in selected contexts, can support tumor localization and molecular subtyping from a single blood draw.^1^, ^2^

**FIGURE 1:**
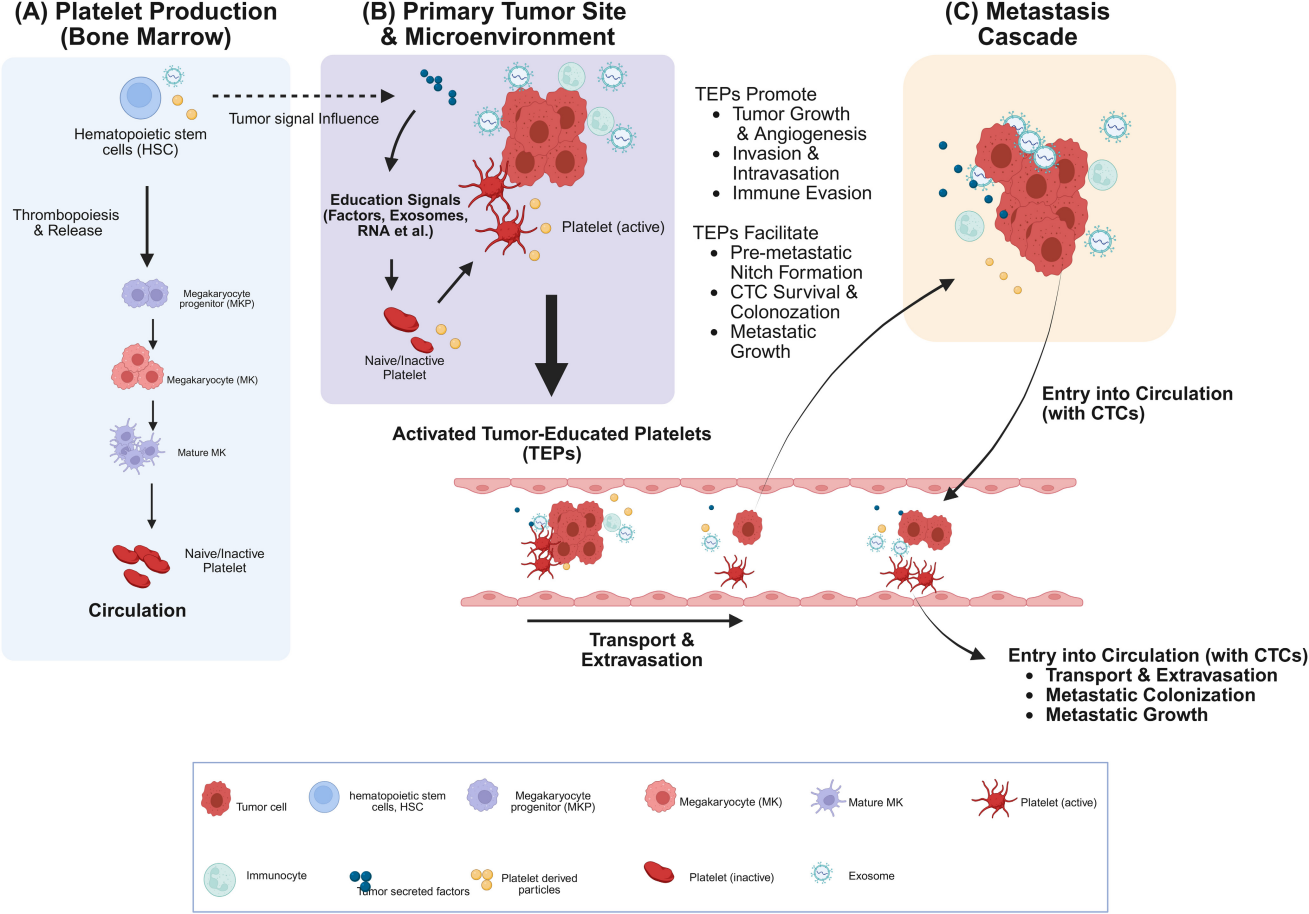
The crosstalk between platelets and cancer cells in tumor progression and metastasis. (A) Platelet production (bone marrow). In the bone marrow, hematopoietic stem cells (HSCs) undergo thrombopoiesis, differentiating through megakaryocyte progenitors (MKPs) and mature megakaryocytes (MKs) to produce naive, inactive platelets that are released into circulation. Note that tumor‐derived signals may influence this production process. (B) Primary tumor site and microenvironment. At the primary tumor site, naive platelets interact with the tumor microenvironment. “Education signals,” including tumor‐secreted factors, exosomes, and RNA transfer, transform these platelets into activated tumor‐educated platelets (TEPs). (C) Metastasis cascade. TEPs are instrumental in promoting metastasis. They facilitate primary tumor growth, angiogenesis, invasion, intravasation, and immune evasion. Furthermore, TEPs aid in the formation of a pre‐metastatic niche and support circulating tumor cells (CTCs). By binding to CTCs, TEPs protect them during transport in the circulation, assist in extravasation, and ultimately support metastatic colonization and growth at distant sites (Created with BioRender.com). CTC, circulating tumor cell; HSC, hematopoietic stem cell; MK, megakaryocyte; MKP, megakaryocyte progenitor; TEP, tumor‐educated platelet.

TEPs' promise reflects evolving biology. Once cast as passive “circulating sponges” for tumor‐derived RNA, platelets are now recognized—as summarized in Figure 1—to do far more.^3^ In experimental models and small patient cohorts, platelets have been shown to scavenge and internalize extracellular DNA, including circulating tumor DNA (ctDNA), supporting an emerging role in cell‐free DNA homeostasis by clearing nucleic acids from plasma.^3^ This dual uptake of RNA and DNA, coupled with intact spliceosomes capable of signal‐dependent pre‐mRNA splicing,^4^, ^5^ provides a mechanistic basis for a multi‐omic platelet readout^2^; whether and to what extent the DNA component will translate into incremental diagnostic performance remains to be established.^3^

Yet translation has stalled on reproducibility. External‐validation studies report limited generalizability of discovery‐phase classifiers and sensitivity to center‐specific (“hospital‐of‐origin”) effects,^6^ with reduced diagnostic accuracy in prospective, higher‐risk cohorts.^7^ Mechanistic data further show that subtle pre‐analytical variation—especially leukocyte/erythrocyte carryover during platelet isolation—distorts the platelet transcriptome and can obscure true biological signal, underscoring the need for rigorous control and harmonization.^8^, ^9^ Together, these data argue that robust standardization is a non‐negotiable foundation for progress.

Against this backdrop, we synthesize evidence from a comprehensive literature review (2010–2025/search strategy and selection criteria are summarized in Box 1) to guide the field from promise to practice. We first dissect the paradox—high discovery‐phase accuracy versus failed replication—to identify failure modes that standard operating procedures (SOPs) must preempt. We then position TEPs within the liquid‐biopsy ecosystem to define a distinct clinical niche and synergies with ctDNA and other analytes.^10^ Finally, we outline a practical roadmap spanning universal SOPs, definitive clinical‐trial designs, and multi‐omics integration for clinical implementation.

BOX 1 **Search Strategy and Selection Criteria.**

We identified references for this Review by searching PubMed and Web of Science for articles published between January 1, 2010, and April 2025, and by consulting the authors' own files. Search terms included “tumor‐educated platelets,” “platelet RNA,” “platelet sequestration,” “liquid biopsy,” “cancer diagnostics,” and “pDNA.” We largely selected studies from the past 5 years (2020–2025) to capture the most recent advances in standardization and multi‐omics, but also included highly regarded older publications seminal to the field. We prioritized articles that provided mechanistic insights, validation cohorts, or standardization protocols. There were no language restrictions, though the search focused on English‐language publications.

## 2 DIAGNOSTIC PERFORMANCE OF TEPs: A CRITICAL APPRAISAL

TEP‐based assays have been evaluated across many solid tumors and often distinguish patients with cancer from healthy controls, frequently achieving high AUCs (Table 1). Yet this promise is tempered by inter‐study heterogeneity and several failed external validations.^6^, ^7^ Diagnostic performance is strongly shaped by standardization rigor, disease stage, and tumor–hematopoietic interactions.^19^, ^20^

**TABLE 1:** Summary of representative studies on TEPs for cancer detection.

Cancer type	TEP biomarker(s) and method	Sample size (cases/controls)	Sensitivity	Specificity	AUC (ROC)	Study design (year)	Classification	Reference
Lung (NSCLC)—localized and metastatic	Spliced platelet RNA panel (thromboSeq RNA‐seq)	Locally advanced: 53 NSCLC/53 controls Metastatic: 245 NSCLC/273 controls	N/A	N/A	0.89 (localized); 0.94 (metastatic)	Multicenter case–control (2017)	[D, IV]	Best et al.^11^
Lung (NSCLC)—early to late stages	881‐gene platelet RNA panel (thromboSeq RNA‐seq); high specificity classifier optimized for specificity	466 NSCLC/410 controls (training); 558 independent validation samples	65% (overall NSCLC) (47% for stage I–III)	94% (controls)	0.88	Multicenter case–control; discovery + validation (2023)	[D, EV]	Antunes‐Ferreira et al.^12^
Lung (NSCLC)—mixed stages	Various mRNA, lncRNA, circRNA, snRNA markers	Meta‐analysis: 7858 lung cancer/6632 controls (pooled from 10 studies)	80% (pooled)	69% (pooled)	0.85 (SROC)	Systematic review and meta‐analysis of TEP diagnostic studies in lung cancer (2023)	[MA]	Wiyarta et al.^13^
Pan‐cancer (18 types)—early detection screening	Platelet spliced RNA pan‐cancer signature (thromboSeq) with machine learning	1096 cancer patients (stage I–IV)/146 asymptomatic controls (discovery and blinded validation)	64% (overall, all stages), 46%–72% (range across stage I–IV)	99% (asymptomatic controls)	– (high specificity threshold)	Multicenter case–control; blinded validation (2022)	[D, EV]	^a^In 't Veld et al.^2^
Breast cancer—early to advanced	Platelet mRNA RNA‐seq; PSO‐SVM and elastic net classifiers	266 breast cancer/212 controls (training); 37/36 (external validation)	85% (internal validation AUC) ~0% effectively in external test (AUC 0.55)	85% (internal, implied by AUC) ~54–55% (external)	0.85 (internal) 0.55 (external)	Multicenter case–control; external validation failed (2023)	[D, EV]	Liefaard et al.^6^
Nasopharyngeal cancer—early detection	Platelet miR‐34c‐3p + miR‐18a‐5p (qPCR assay)	54 NPC/36 controls (approx.)	94% (miR‐34c, positivity) 88% (miR‐18a); 95% (combined AUC)	90%–95% (estimated at optimized threshold)	0.95 (combined)	Single‐center case–control (2019)	[D, EV]	Wang et al.^14^
Colorectal and GI cancer—mixed stages	Platelet 2‐gene mRNA signature (*CDK1* up, *HSPA5* down) via RNA‐seq meta‐analysis	3 pooled GI cancer cohorts (775 patients/340 controls total)	79%–88% (estimated, per nomogram high risk cut‐off)	~85% (estimated)	~0.90 (nomogram model)	Retrospective bioinformatic meta‐analysis; small validation (2023)	[D]	Jiang et al.^15^
Ovarian cancer—early versus late	Platelet protein panel (VEGF, PDGF, PF4, CTAP3, TSP‐1) + count/volume (PLS‐DA model)	139 ovarian cancer (47 early/92 late)/98 benign and healthy	83% (early stage I–II) 96% (late stage III–IV)	76% (early) 88% (late)	0.83 (early)	Single‐center case–control (2018)	[D]	Lomnytska et al.^16^
Sarcoma (all stages)	TEP RNA‐seq signature	115 Sarcoma/100 controls	70%	80%	0.80	Case–control (2020)	[D]	Heinhuis et al.^17^
Glioma versus controls	TEP RNA‐seq (thromboSeq)	206 Glioma/354 controls	N/A	N/A	0.87	Multicenter case–control (2020)	[D, IV]	Sol et al.^18^

*Note*: Data interpretation flags: To contextualize performance metrics, studies are annotated with the following design indicators: [D] discovery/training cohort; [IV] internal validation (e.g., cross‐validation); [EV] external/independent validation; [MA] meta‐analysis. Performance metrics (AUC, sensitivity) from [D] studies may be optimistic compared to [EV] cohorts.

Abbreviations: AUC, area under the curve; *CDK1*, cyclin‐dependent kinase 1; circRNA, circular RNA; CTAP3, connective tissue‐activating peptide 3; D, discovery; EV, external validation; GI, gastrointestinal; *HSPA5*, heat shock protein family A (Hsp70) member 5; IV, internal validation; lncRNA, long non‐coding RNA; MA, meta‐analysis; miR, microRNA; mRNA, messenger RNA; N/A, Not Applicable; NPC, nasopharyngeal carcinoma; NSCLC, non‐small cell lung cancer; PDGF, platelet‐derived growth factor; PF4, platelet factor 4; PLS‐DA, partial least squares‐discriminant analysis; PSO‐SVM, particle swarm optimization‐support vector machine; qPCR, quantitative polymerase chain reaction; RNA‐seq, RNA sequencing; ROC, receiver operating characteristic; snRNA, small nuclear RNA; SROC, summary receiver operating characteristic; TEP, tumor‐educated platelet; TSP‐1, thrombospondin‐1; VEGF, vascular endothelial growth factor.

^a^ In 't Veld et al. pan‐cancer TEP study (Cancer Cell 2022): Validation series included 1096 cancer patients, 146 asymptomatic controls, and 333 symptomatic controls. At a threshold optimized to 99% specificity in asymptomatic controls (95% CI 95–100), overall sensitivity was 64% with stage‐specific detection of 46%, 47%, 54%, and 72% for stages I–IV (*n* = 65, 112, 175, and 617, respectively; 61% for unknown stage, *n* = 127). Cancer‐specific sensitivities were heterogeneous (e.g., ~40% for breast cancer vs. 92% for prostate cancer, 11/12 cases). Specificity decreased to 78% in symptomatic controls (*n* = 333; 95% CI 73–82), illustrating substantial spectrum effects and an increased false‐positive burden in real‐world referral populations.

### 2.1 The impact of standardization: Lessons from NSCLC and breast cancer

Technical confounders are best illustrated by contrasting non‐small cell lung cancer (NSCLC) with breast cancer. In NSCLC, thromboSeq (platelet RNA‐seq plus machine learning) showed high accuracy across stages (AUC 0.89 with 81% accuracy in early‐stage validation; AUC 0.94 with 88% accuracy in late‐stage validation).^19^ A multicenter, 881‐gene signature achieved AUC 0.88 in independent validation, with tunable high‐sensitivity/high‐specificity operating modes enabled by predefined QC and harmonized processing.^1^, ^12^

By contrast, in breast cancer, a classifier with an internal AUC 0.85 fell to ~0.55 in a blinded external cohort; hospital‐of‐origin explained ~19% of expression variance—classic batch effects that obscure biology.^6^ Even low‐level leukocyte/erythrocyte carryover and protocol differences can shift platelet transcriptomes, underscoring the need for universal SOPs and quantitative QC metrics.^8^, ^21^ A 2023 meta‐analysis of lung‐cancer TEP studies (10 studies; 7858 cases; 6632 controls) reported pooled AUC 0.85 and specificity 0.69, with substantial heterogeneity by stage, control selection, assay modality, and RNA type—dimensions closely tied to standardization.^13^ Together, these data make rigorous protocol standardization non‐negotiable for clinical translation.^6^, ^12^ A recent lung cancer‐focused review similarly concludes that TEPs are attractive liquid‐biopsy candidates but remain investigational, with substantial unmet needs in assay standardization, prospective validation, and integration with other biomarkers in thoracic oncology.^22^

### 2.2 Performance across stages: The potential for early detection

Platelets may integrate tumor‐derived signals over time, supporting sensitivity in early‐stage disease as emphasized in early‐biomarker reviews.^23^ In multi‐cancer early detection (MCED), In 't Veld et al. profiled 18 cancers in a validation cohort comprising 1096 cancer patients, 146 asymptomatic controls, and 333 symptomatic controls. At a threshold tuned to 99% specificity in asymptomatic controls (146/146; 95% CI 95–100), overall sensitivity was 64%, with stage‐specific detection rates of 46% for stage I (*n* = 65), 47% for stage II (*n* = 112), 54% for stage III (*n* = 175), and 72% for stage IV (*n* = 617); tumor‐of‐origin was correctly identified in >80% for several cancers, but per‐cancer sensitivities were heterogeneous, ranging from approximately 40% for breast cancer to 92% for prostate cancer (11/12 cases).^2^

Notably, TEPs can capture earlier‐stage signals where ctDNA often underperforms.^24^ Recent work highlights alternative splicing events (e.g., *TMEM219*) that discriminate stage I NSCLC—including ground‐glass opacities.^25^ —and lncRNA‐enriched signatures (e.g., *STARD4‐AS1*, *ELOA‐AS1*) that improved classification in small cohorts; these markers require blinded, prospective validation in screening‐representative populations.^25^, ^26^

Recent primary data have further expanded this non‐coding landscape to include circular RNAs (circRNAs). For instance, Campolo et al. identified a distinct platelet‐derived circRNA signature in patients with gastroenteropancreatic neuroendocrine tumors (GEP‐NETs), suggesting that circRNAs—alongside mRNAs and miRNAs—convey tumor‐type‐specific diagnostic information and warrant integration into multi‐omic TEP panels.^27^ In urologic oncology, discovery‐set data in renal cell carcinoma show promising discrimination linked to angiogenesis biology but remain sample‐limited.^28^ In prostate cancer, tumor‐related transcripts (e.g., *PCA3*) are detectable in platelets, yet platelet RNA alone may not reliably capture early disease.^29^ Overall, feasibility coexists with mandates for standardized pipelines and prospective replication.^28^, ^29^ Recently described pDNA adds a potentially more stable analyte: internalized DNA appears nuclease‐protected in vitro,^3^, ^30^ and *BRAF* driver mutations have been detected in pDNA from patients with high‐risk sessile serrated colonic lesions—sometimes at higher allele abundance than matched plasma cfDNA in small discovery cohorts^3^—suggesting that pDNA could improve sensitivity when tumor burden and cfDNA shedding are minimal. These observations remain preliminary and require independent replication in larger, clinically annotated series.

### 2.3 Dissecting heterogeneity: Biological versus technical factors

**FIGURE 2:**
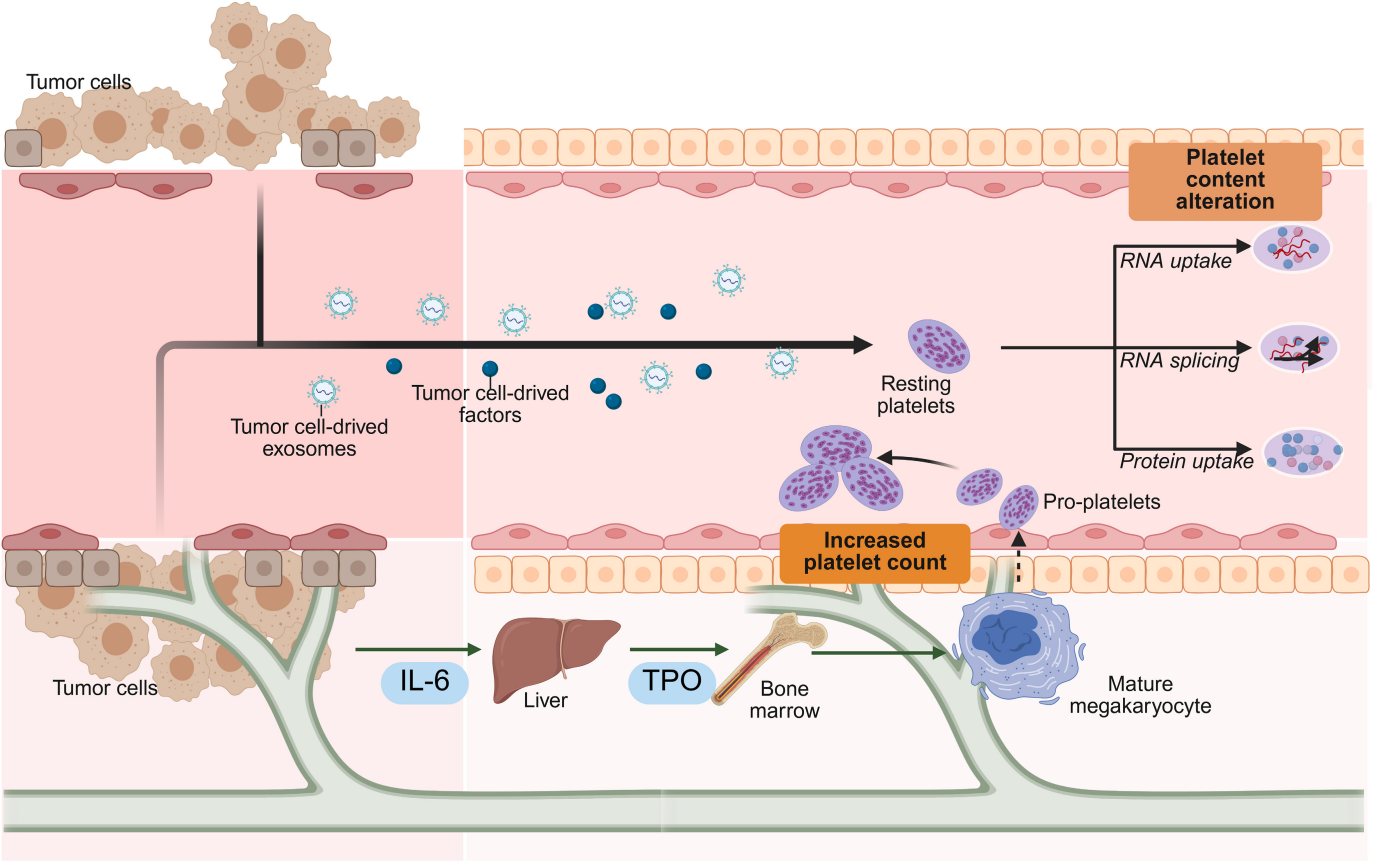
Mechanisms of tumor‐platelet crosstalk: promoting cancer via local activation and systemic reprogramming. Tumor cells shed exosomes and soluble factors that enter the circulation and are incorporated by platelets, altering platelet content through RNA uptake, intraplatelet RNA splicing, and protein uptake. In parallel, tumor‐derived interleukin‐6 (IL‐6) drives hepatic thrombopoietin (TPO) production, which promotes megakaryopoiesis in the bone marrow and increases platelet output from mature megakaryocytes via pro‐platelet formation. Together, these processes expand platelet numbers and reprogram their molecular cargo, generating tumor‐educated platelets in the bloodstream. (Created with BioRender.com). IL‐6, interleukin‐6; RNA, ribonucleic acid; TPO, thrombopoietin.

Performance variability reflects both technical and biological heterogeneity. Technically, centrifugation schemes, extraction chemistries, library/sequencing protocols, and normalization can shift platelet‐RNA profiles and downstream classifiers; multicenter breast‐cancer evaluations showed strong site effects and failed external validation despite solid internal AUCs, reinforcing the need for harmonized pre‐analytics and cross‐site calibration.^6^, ^31^ Deconvolution analyses reveal substantial non‐platelet read content and activated‐platelet/inflammatory programs that can mask or mimic tumor‐dependent biology without computational correction.^32^ Biologically, platelet “education” likely varies by tumor type, histology, and vascular dynamics. Recent evidence highlights that pathways such as STAT3 and epithelial–mesenchymal transition (EMT) provide representative examples of signaling axes that modulate tumor plasticity and therapy response.^33^ In contrast, Figure 2 depicts a more general schematic of tumor‐platelet crosstalk, summarizing how tumor‐derived exosomes, soluble factors, and cytokine‐driven megakaryopoiesis cooperate to reprogram platelet number and cargo.^34^ In the MCED cohort, detection rates differed markedly across cancers, indicating genuine tumor‐to‐platelet diversity.^2^ Aggressively angiogenic tumors (e.g., renal cell carcinoma, glioblastoma) may shed more platelet‐activating mediators and vesicles, amplifying TEP signals, whereas indolent or encapsulated tumors interact less with the circulation until later stages.^34^ Thus, the variability observed by In 't Veld et al. reflects tumor‐hematopoietic coupling at least as much as platform limitations.^2^

Collectively, current data indicate that TEP performance is not uniform across tumor types. Tumors with intense vascular remodeling and tumor‐hematopoietic crosstalk, such as NSCLC, glioma, ovarian and other gastrointestinal malignancies, nasopharyngeal carcinoma, and sarcoma, tend to outperform, with single‐cancer studies frequently reporting AUCs in the ~0.80–0.95 range (Table 1). By contrast, breast cancer has emerged as a relative underperformer: platelet‐RNA classifiers with promising internal performance (AUC ≈ 0.85) have shown markedly reduced discrimination in external cohorts (AUC ≈ 0.55),^6^ and breast cancer exhibited among the lowest per‐cancer sensitivities (~40%) in the pan‐cancer MCED study, whereas prostate cancer was at the opposite end of the spectrum (~90% sensitivity).^2^ These patterns support a model in which tumor type‐specific biology and tumor‐hematopoietic coupling amplify or constrain the diagnostic signal, superimposed on the technical sources of heterogeneity outlined above.

## 3 TEPs IN THE CONTEXT OF THE LIQUID BIOPSY ECOSYSTEM

To accurately contextualize the diagnostic performance and the sources of heterogeneity described above, TEPs should be viewed alongside circulating tumor DNA (ctDNA), circulating tumor cells (CTCs), and tumor‐derived extracellular vesicles/exosomes. Each modality samples a different biological layer and thus offers distinct trade‐offs in analytical sensitivity, specificity, information content, and workflow maturity (Table 2).^10^

**TABLE 2:** Qualitative comparison of liquid biopsy platforms in cancer diagnostics.

Feature	Tumor‐educated platelets (TEPs)^1^, ^2^, ^6^, ^12^, ^35^, ^36^, ^37^, ^38^	Circulating tumor DNA (ctDNA)^39^, ^40^, ^41^, ^42^, ^43^, ^44^, ^45^, ^46^	Circulating tumor cells (CTCs)^47^, ^48^, ^49^, ^50^, ^51^	Extracellular vesicles (EVs)/exosomes^52^, ^53^, ^54^, ^55^, ^56^, ^57^
Biomarker content	Spliced mRNA, non‐coding RNAs, proteins. In experimental studies, platelets also contain sequestered nuclear DNA fragments (pDNA) that can harbor tumor‐specific mutations, CNVs, and methylation patterns; the robustness and prevalence of this signal in clinical populations remain under active investigation. Together, these analytes provide a potentially holistic readout of tumor–host interactions.	Fragmented tumor DNA (mutations, CNVs, methylation). Direct genetic alterations from tumor cells. No protein content.	Intact tumor cells (can assess DNA, RNA, protein, morphology). Very high specificity—a direct tumor cell.	Membrane‐bound vesicles containing tumor‐derived proteins, DNA, mRNA, and miRNA. Molecular snapshot of tumor cells' content, but mixed with normal EVs.
Sensitivity (early disease)	High potential. Time‐integrated signal accumulation is advantageous for early detection. Early proof‐of‐concept data suggest that pDNA signals may exceed plasma cfDNA in some premalignant lesions, but this observation is based on small cohorts and requires independent replication.	Variable; often low in stage I (many false negatives). For example, <50% detection in some stage I cancers (due to low tumor DNA fraction). Improves with tumor size.	Extremely low in early stages (few or zero CTCs in stage I/II). Better in metastatic disease but still may miss cases.	Moderate; some reports of exosomal markers in early cancer, but large variability. Can be more sensitive than ctDNA in certain contexts, but data still emerging.
Specificity	Can be very high (94%–99%) in controlled studies. Vulnerable to confounding by inflammation and other non‐malignant conditions	Very high if tumor‐specific mutations are detected (background low). False positives rare, aside from clonal hematopoiesis causing “tumor‐like” mutations.	Essentially 100% when a CTC is identified (tumor cell presence is pathological). Misidentification (rare) possible if a normal cell is mistakenly counted.	High in principle, but differentiating tumor EVs from normal EVs is challenging, requiring panel‐based assays to achieve specificity
Sample handling	Requires careful, standardized centrifugation to prevent activation and contamination. Internalized pDNA appears highly stable and protected from nucleases in mechanistic studies, but its behavior in routine clinical workflows remains to be defined.	Simple blood draw. Stabilization tubes (e.g., Streck) often used to stabilize DNA and prevent cell lysis. If standard EDTA tubes are used, rapid plasma processing is required to avoid genomic DNA contamination. Plasma DNA extraction straightforward.	Special collection tubes and processing to enrich rare cells (immunomagnetic capture, filtration, etc.). Technically involved.	Blood draw, then ultracentrifugation or immunoisolation for exosomes. More labor‐intensive than plasma DNA prep.
Assay methods	RNA‐seq, qPCR, ddPCR, plus analysis of pDNA. Standardized bioinformatic pipelines are essential to control batch effects.	Digital PCR or next‐generation sequencing (NGS) for mutations; targeted panels or whole‐genome/epigenome assays. Well‐developed protocols.	Microscopy or flow cytometry‐based counting; downstream DNA/RNA sequencing on isolated cells possible (single‐cell analysis).	NGS for exosomal RNA/DNA; proteomic assays (e.g., immunoassays or mass spec) for exosome proteins. Some microfluidic chip technologies.
Key strength	Time‐integrated signal with high potential for early detection. May act as a relatively protected reservoir for DNA/RNA, although the clinical impact of this feature is not yet established. Reflects tumor phenotype.	Acquisition of viable cells. Unparalleled for studying	Metastatic mechanisms and ex‐vivo drug response testing	Diverse molecular cargo (incl. proteins). Important for studying intercellular communication
Cost	Currently high (RNA‐seq). Potentially moderate with targeted kits. Uses standard lab equipment (centrifuge, PCR)—accessible.	Ranges from moderate (PCR single‐gene) to high (broad NGS panel or multi‐cancer test). Specialized reagents needed, but many providers exist.	High per test (due to labor and low throughput). Instruments like CellSearch are expensive and not widely available outside major centers.	Potentially high—isolation and analysis of exosomes not yet standardized, requiring advanced equipment. Costs expected to drop with new isolation technologies.
Clinical use status	Investigational. No FDA‐approved tests to date.	Established in clinical practice. Several FDA‐approved companion diagnostic (CDx) tests exist. MCED tests are available as LDTs.	Limited. FDA‐cleared for prognosis in metastatic cancer (CellSearch) but not widely used to guide therapy.	Investigational. Niche use‐cases exist (e.g., urine exosome test for prostate cancer risk) but not in general oncology.
Primary clinical niche (best‐in‐class use case)	Early detection and phenotyping: integrating weak systemic signals for screening and tumor localization (time‐integrated sensor).	MRD and genotyping (CDx): monitoring tumor burden/recurrence and guiding targeted therapy via specific mutations.	Ex vivo modeling: functional testing (e.g., organoids, drug sensitivity) and studying metastatic mechanisms via intact cells.	Intercellular signaling: analyzing complex tumor‐host communication and multi‐omics in low‐shedding tumors.

Abbreviations: CDx, companion diagnostic; cfDNA, cell‐free DNA; CNV, copy number variation; CTC, circulating tumor cell; ctDNA, circulating tumor DNA; ddPCR, droplet digital polymerase chain reaction; EDTA, ethylenediaminetetraacetic acid; EV, extracellular vesicle; FDA, Food and Drug Administration; LDT, laboratory developed test; MCED, multi‐cancer early detection; miRNA, microRNA; MRD, minimal residual disease; mRNA, messenger RNA; NGS, next‐generation sequencing; PCR, polymerase chain reaction; pDNA, platelet‐sequestered DNA; qPCR, quantitative polymerase chain reaction; RNA‐seq, RNA sequencing; TEP, tumor‐educated platelet.

### 3.1 Sensitivity and specificity: A dynamic balance

Achieving credible early‐stage detection requires exceptional sensitivity without compromising specificity. ctDNA assays often struggle at presentation because tumor DNA fractions in plasma are frequently near or below technical limits in stage I–II disease, despite substantial progress with tumor‐informed, ultra‐deep approaches.^24^, ^58^

By contrast, TEPs may retain earlier visibility due to two key mechanisms. First, platelets continuously sample tumor‐derived cues over their ~7–10‐day lifespan, effectively integrating signals over time. Second, Murphy et al. recently provided mechanistic evidence that platelets can internalize extracellular DNA, including tumor‐derived fragments, and that internalized DNA is resistant to degradation by exogenous DNases, supporting the emerging concept that pDNA may act as a relatively protected, time‐integrated reservoir of tumor DNA.^3^ These experiments were performed in limited cohorts under highly controlled conditions, and potential confounders—such as platelet–leukocyte aggregates, residual buffy‐coat contamination, and ex vivo DNA uptake during sample handling—must be rigorously excluded in future work before concluding that pDNA will confer clinically meaningful sensitivity gains. Independently of pDNA, a large pan‐cancer study using platelet RNA classifiers achieved 99% specificity in asymptomatic controls (146/146; 95% CI 95–100) with overall sensitivity of 64% and approximately 50% detection across stage I–III disease.^2^ However, when applied to symptomatic controls with non‐malignant cardiovascular, benign mass, or inflammatory conditions, specificity dropped to 78% (*n* = 333; 95% CI 73–82), implying substantial false‐positive rates and downstream imaging/biopsy burden if such assays are deployed in real‐world referral populations without additional triage or stricter thresholds.^2^

Specificity can be high across platforms with disciplined assay design. ctDNA tumor‐informed platforms report ≥99% specificity at parts‐per‐million detection limits, though interpretation must explicitly address clonal hematopoiesis (CHIP) to avoid false positives.^58^, ^59^ TEP profiles can approach high specificity in low‐inflammation cohorts, but platelet activation/inflammation signatures and non‐platelet RNA carryover can erode specificity unless bioinformatically controlled.^2^, ^32^

### 3.2 Molecular information yield and localization

ctDNA excels at genomic/epigenomic profiling (mutations, copy number, methylation), supporting therapy selection and MRD monitoring.^60^ TEPs, in contrast, can deliver multi‐omic readouts—transcriptomic (splicing, lncRNAs), proteomic, and lipidomic states—reflecting tumor–host crosstalk and systemic biology beyond the genome.^61^, ^62^ Notably, platelet RNA signatures can infer tumor origin; in the 2022 pan‐cancer analysis, site‐of‐origin was correctly assigned in >80% for several tumor types.^2^, ^60^, ^61^, ^63^

### 3.3 Logistical feasibility and maturity

ctDNA pipelines are comparatively mature and already FDA‐approved for multiple companion‐diagnostic indications. In contrast, TEP analysis requires stricter pre‐analytics (controlled centrifugation to isolate unactivated platelets, minimization of leukocyte carryover) and harmonized library/normalization steps.^1^, ^62^ While early work emphasized immediate processing, stability studies indicate that platelet RNA signatures change minimally over ~48–72 h under defined conditions, offering practical flexibility for multicenter workflows.^18^, ^20^ As of mid‐2025, no TEP‐based diagnostic has received FDA marketing authorization; by comparison, no MCED blood test (of any analyte) has FDA authorization to date, and CTC detection has one FDA‐cleared platform (CellSearch).^10^, ^64^

### 3.4 The imperative for integrated approaches

Given their complementary biology, multi‐analyte strategies are compelling. Proof‐of‐concept MCED work combining ctDNA mutations with protein markers demonstrated improved detection/localization.^65^ Similar integrated designs—for example, merging TEP multi‐omics with ctDNA and exosomal proteins—are logical next steps; emerging reviews and pilot studies endorse such combinatorial approaches to balance sensitivity and specificity while mitigating single‐analyte blind spots (Table 3).^20^, ^68^

**TABLE 3:** Practical decision matrix: when to prefer ctDNA, TEPs, or combined testing.

Feature	ctDNA dominant^66^, ^67^	TEP dominant^2^, ^18^	Combined strategy^67^
Primary utility	Genotyping and MRD (mutation tracking)	Screening and localization (tissue‐of‐origin)	Diagnostic resolution (max sensitivity/specificity)
Optimal clinical scenario	Therapy selection (e.g., *EGFR*); high‐shedding tumors (metastatic)	Early detection (stage I); low‐shedding tumors (e.g., GBM); cancer of unknown primary (CUP)	Indeterminate pulmonary nodules; high‐stakes diagnostic triage
Pre‐analytics	Flexible (stabilization tubes allowed)	Strict (2‐step spin, immediate lysis)	Complex (dual workflows required)
Turnaround time	Fast (<7 days; PCR/panel)	Moderate (7–14 days; RNA‐seq)	Slow (cumulative analysis)
Relative cost	$–$$ (targeted assays)	$$$ (whole transcriptome)	$$$$ (additive costs)

Abbreviations: ctDNA, circulating tumor DNA; CUP, cancer of unknown primary; *EGFR*, epidermal growth factor receptor; GBM, glioblastoma multiforme; MRD, minimal residual disease; PCR, polymerase chain reaction; RNA‐seq, RNA sequencing; TEP, tumor‐educated platelet.

## 4 TRANSLATIONAL POTENTIAL: CLINICAL APPLICATIONS OF TEP ASSAYS

The unique biology of TEPs equips them to address unmet needs from population screening to individualized treatment monitoring.^1^ Specifically, TEP implementation aims to improve clinical outcomes through three key avenues: (1) enabling a “stage shift” to curative diagnoses via sensitive early detection, (2) reducing the morbidity of unnecessary invasive procedures through high‐specificity triage of indeterminate lesions, and (3) optimizing therapeutic efficacy by identifying responders to immunotherapies and detecting minimal residual disease (MRD) earlier than current standards.

### 4.1 Screening and early detection

For population screening, approximately 99% specificity is essential to minimize downstream harms; a 2022 pan‐cancer platelet‐RNA MCED study demonstrated ~99% specificity with stage‐stratified detection, indicating feasibility at such thresholds.^2^

To bridge the gap between discovery‐phase metrics and clinical utility, it is crucial to interpret performance in the context of disease prevalence.^69^ In a typical balanced case–control study (prevalence 50%), a TEP assay with 80% sensitivity and 90% specificity yields an impressive positive predictive value (PPV) of 89%. However, applied to a real‐world high‐risk lung cancer screening setting with a prevalence of ~1.5% (15 cancers per 1000 individuals), that same 90% specificity results in a PPV of only ~11% (12 true positives vs. ~99 false positives), creating an unacceptable burden of unnecessary follow‐up procedures.^69^, ^70^ Even maximizing specificity to 99% yields a PPV of approximately 55%. This mathematical reality underscores why our roadmap prioritizes validating TEPs in intended‐use cohorts with stringent specificity thresholds (≥99%) rather than relying solely on AUCs derived from artificial case–control datasets.^70^, ^71^

Low‐dose computed tomography (LDCT) reduces lung‐cancer mortality but carries high false‐positive rates, highlighting the potential value of adjunctive molecular triage.^72^ Organ‐focused TEP signatures for early NSCLC—including alternative splicing events such as *TMEM219*—may refine management of indeterminate pulmonary nodules detected by LDCT.^25^ Consequently, TEPs warrant evaluation as add‐on tests to raise the PPV of imaging‐based screening while preserving stringent specificity.^2^, ^25^, ^72^

However, the successful deployment of such powerful screening tools requires addressing the patient experience and ethical landscape. Chen et al. demonstrate that structured decision tools are essential to improve screening acceptance and ensuring patients comprehend the complexities of multi‐cancer testing.^73^ Furthermore, the potential for detecting malignancy without clear localization—effectively creating a “molecular” cancer of unknown primary (CUP)—raises profound ethical concerns regarding over‐diagnosis and patient autonomy that must be navigated through robust counseling frameworks and clear management protocols.^74^

### 4.2 Dynamic monitoring of treatment response and MRD

Beyond diagnosis, TEPs are promising for longitudinal monitoring and MRD. Unlike the short half‐life of circulating tumor DNA (ctDNA), platelets (7–10‐day lifespan) can, in principle, harbor tumor‐derived nucleic acids and may smooth perioperative or other transient nadirs; under defined conditions, platelet RNA profiles remain stable for 48–72 h, enabling multicenter workflows.^20^ Recent work by Li et al. further illustrates how such predictive platelet biomarkers can be practically embedded in longitudinal care to track treatment trajectories.^75^ This advantage is salient in low‐ or intermittent‐shedding tumors: in glioblastoma, TEP‐based classifiers have distinguished true progression from pseudoprogression and tracked disease dynamics.^18^, ^76^ In renal cell carcinoma (RCC)—a canonical low‐ctDNA‐shedding tumor—platelet RNA panels show early feasibility for response assessment and relapse surveillance.^28^, ^77^ Signals in sarcomas and early‐stage NSCLC further support sensitive longitudinal monitoring, though larger, blinded, prospective studies are required for adoption.^17^, ^25^

### 4.3 Predictive biomarkers and molecular profiling

When tissue is unavailable, TEP profiling can recover molecular features—for example, *EGFRvIII* in glioblastoma and *PCA3*/*KLK3* in prostate cancer—supporting the presence of tumor‐related transcripts in platelets.^29^, ^37^ Specifically, regarding sensitivity and specificity for molecular alterations, TEPs show distinct advantages in detecting RNA‐level structural variants.^20^ For example, TEP RNA‐seq has demonstrated >90% specificity for detecting the *EGFRvIII* splice variant in glioblastoma patients, a target that is challenging for plasma DNA assays due to its non‐genomic nature.^18^, ^78^, ^79^ Although *AR‐V7* is typically measured in circulating tumor cells (CTCs) or exosomal RNA, exploratory data suggest platelet RNA‐based detection is feasible and merits validation.^80^, ^81^ Beyond genotype, TEPs encode activation and inflammatory programs that reflect microenvironmental states not captured by tumor DNA.^32^ In immuno‐oncology, platelet PD‐L1 (pPD‐L1) has predicted response to PD‐1 blockade in NSCLC and other cancers, sometimes outperforming tissue PD‐L1.^82^, ^83^ Beyond immune checkpoints, TEPs are conceptually positioned to offer a window into therapy resistance mechanisms by reflecting dynamic, treatment‐induced adaptations in tumor and host biology. Emerging omics studies of therapy resistance in cancer more broadly highlight that complex metabolic and signaling adaptations under drug pressure can be systematically deconvolved to reveal targetable vulnerabilities,^84^, ^85^ supporting the broader rationale for developing systemic liquid‐biopsy readouts—including TEP‐based assays—to monitor evolving resistance patterns.

Pathway‐level analyses implicate hypoxia and related stress programs in shaping circulating signals, motivating studies of TEP expression modules (e.g., hypoxia/IFN‐γ) as predictors of checkpoint‐inhibitor benefit.^86^ Collectively, TEPs provide phenotype‐rich information complementary to ctDNA, spanning tumor genotype and dynamic tumor‐immune physiology.^32^, ^82^, ^83^, ^86^

These applications—from early detection and MRD monitoring to predictive biomarkers—remain unrealized; closing the gap will require a rigorous, standardized translational strategy to overcome technical and biological barriers.

## 5 A ROADMAP FOR CLINICAL IMPLEMENTATION: OVERCOMING CRITICAL BARRIERS

Realizing the clinical applications outlined above—from MCED to sensitive minimal residual disease (MRD) monitoring—is not inevitable; it is a design problem. For TEP assays to advance from concept to reliable diagnostics, both technical and biological heterogeneity must be addressed through common SOPs, multicenter validation, and prospective utility studies.^87^

### 5.1 Pre‐analytical control: The most immediate vulnerability

**FIGURE 3:**
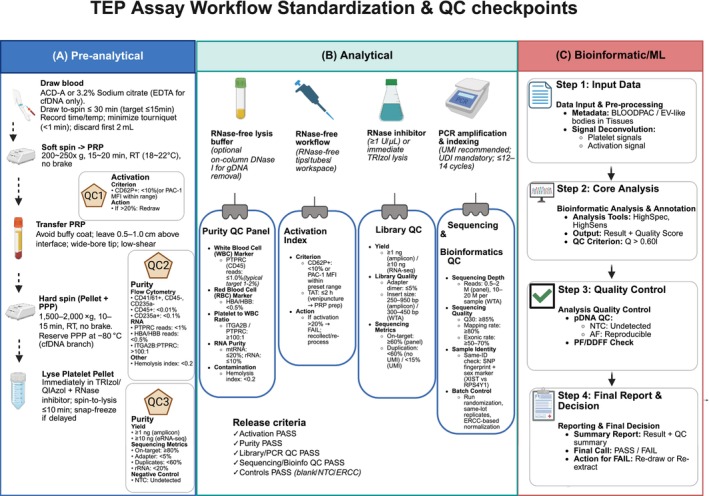
Standardized workflow and quality control (QC) checkpoints for the tumor‐educated platelet (TEP) assay. (A) Pre‐analytical phase. The blood collection and processing protocol is summarized. This includes an initial soft‐spin centrifugation to obtain platelet‐rich plasma (PRP), followed by a hard spin to isolate the platelet pellet and platelet‐poor plasma (PPP). Key QC criteria for platelet activation (QC1), purity by flow cytometry (QC2), and RNA yield and purity (QC3) are specified. (B) Analytical phase. This section details the laboratory workflow, including requirements for an RNase‐free environment and key reagents. It specifies the comprehensive QC panels used to assess sample purity, platelet activation, sequencing library quality, and sequencing run performance. The final release criteria that must be met before proceeding to data analysis are summarized. (C) Bioinformatic/ML phase. The computational pipeline is depicted, beginning with data input and pre‐processing, including the deconvolution of platelet and activation signals. The workflow proceeds through core analysis, a dedicated quality control step for the processed data, and culminates in a final report with a PASS/FAIL decision, guiding whether to re‐draw the sample or re‐extract (Created with BioRender.com). ACD‐A, anticoagulant citrate dextrose solution A; AF, allele frequency; BLOODPAC, blood profiling atlas in cancer; bp, base pair; CD, cluster of differentiation; CD41, cluster of differentiation 41; CD45, cluster of differentiation 45; CD62P+, cluster of differentiation 62P (P‐selectin); CD235a+, cluster of differentiation 235a (Glycophorin A); cfDNA, cell‐free DNA; DDFF, data‐driven filter fail; EDTA, ethylenediaminetetraacetic acid; ERCC, external RNA controls consortium; gDNA, genomic DNA; *HBA*, hemoglobin subunit alpha; *HBA*/*HBB*, hemoglobin subunit alpha/beta; *HBB*, hemoglobin subunit beta; ITGA2B, integrin subunit alpha 2b; M, million; MFI, mean fluorescence intensity; ML, machine learning; mtDNA, mitochondrial DNA; mtRNA, mitochondrial RNA; ng, nanogram; NTC, no‐template control; PAC‐1, antibody binding to activated platelet receptor GPIIb/IIIa; PCR, polymerase chain reaction; pDNA, platelet‐sequestered DNA; PF, Pass Filter; PFP, platelet‐free plasma; PPP, platelet‐poor plasma; PRP, platelet‐rich plasma; *PTPRC*, protein tyrosine phosphatase receptor type C; Q, quality score; QC, quality control; RBC, red blood cell; *RPS4Y1*, ribosomal protein S4 Y‐Linked 1; rRNA, ribosomal RNA; SOP, standard operating procedure; TAT, turnaround time; TEP, tumor‐educated platelet; UDI, unique dual index; UMI, unique Molecular Identifier; WBC, White Blood cell; WTA, whole transcriptome amplification; *XIST*, X‐inactive specific transcript.

Platelets are exquisitely sensitive to ex vivo handling. Anticoagulant choice, venipuncture technique, draw‐to‐process time, centrifugation profile, and temperature all shift activation state and downstream molecular readouts.^62^, ^88^ In a multicenter breast‐cancer study, two TEP classifiers failed blinded external validation; ≈19% of platelet gene‐expression variance was attributable to hospital‐of‐origin, implicating within‐protocol processing differences as major confounders.^6^ Without rigorous pre‐analytics, batch effects can overwhelm true biology.^6^, ^62^ Specifically, the isolation procedure faces a critical dilemma: the trade‐off between purity and quiescence. While rigorous leukocyte depletion is essential—as high‐abundance leukocyte RNA can easily mask the low‐input platelet transcriptome^8^—aggressive purification methods often induce shear stress. This mechanical stress triggers ex vivo activation and splicing alterations that mimic tumor‐educated signatures, thereby confounding the analysis.^89^, ^90^ To resolve this, a universal SOP should codify four essentials (Figure 3).

First, anticoagulant and phlebotomy. EDTA tubes are generally preferred for RNA‐centric workflows because platelet RNA profiles remain relatively stable for ~48–72 h in EDTA at room temperature under thromboSeq‐style conditions, whereas citrate may be preferable for functional/proteomic assays to minimize artifactual activation; tourniquet time should be minimized.^2^, ^20^, ^62^

Second, centrifugation. Apply a defined two‐spin differential centrifugation (soft spin to obtain PRP, followed by a harder spin to pellet platelets) with fixed g‐force, duration, temperature, and brake settings across sites, as used in thromboSeq and recent NSCLC TEP studies.^12^, ^91^

Third, processing and stabilization. Immediately lyse purified platelets in chaotropic RNA buffer to suppress ex vivo transcriptional drift and RNase activity, as standard in platelet RNA‐seq pipelines.^92^

Fourth, stringent quality control. Contamination by leukocytes/erythrocytes introduces exogenous transcripts and must be monitored; multiple groups have detected leukocyte‐derived reads in insufficiently purified “platelet” RNA, arguing for explicit purity checks.^8^ Activation status should be tracked by flow cytometry using canonical markers (CD62P/P‐selectin; integrin αIIbβ3 activation via PAC‐1/fibrinogen) under ISTH SSC guidance.^88^, ^93^ To increase reproducibility, we propose adopting numeric acceptance criteria derived from successful discovery cohorts: for example, rejecting samples exceeding 15%–20% P‐selectin positivity (identifying ex vivo activation)^94^ and enforcing purity thresholds of <1 leukocyte event per 10^6^ platelets (or negligible *PTPRC*/*CD45* read counts in RNA‐seq data).^95^, ^96^ Establishing such quantitative “go/no‐go” metrics is critical to prevent technical noise from masquerading as biological signal.

To convert standardization from aspiration to reality, international ring trials are imperative. Existing liquid‐biopsy consortia provide scaffolds: BLOODPAC has articulated minimum technical data elements (MTDEs) for pre‐analytics and expanded clinical/patient‐context elements to support reproducibility and regulatory‐grade evidence, and the European Liquid Biopsy Society (ELBS) has prioritized harmonized workflows and reporting across Europe.^87^, ^97^ A dedicated TEP Working Group within these consortia could adapt MTDEs for platelet‐RNA assays (e.g., mandated recording of anticoagulant, tube lot, draw‐to‐spin time, spin g‐forces/temperature, and activation/QC metrics), cross‐ship common reference materials, and publish a unified, evidence‐based SOP suitable for prospective, multicenter clinical validation.

Taken together, a consensus SOP anchored in EDTA‐based RNA workflows (or citrate for functional proteomics, as appropriate), tightly specified two‐spin processing with immediate lysis, and mandatory purity/activation QC—tested and refined through BLOODPAC/ELBS‐sponsored ring trials—offers the most credible path to improving signal‐to‐noise, reducing site effects, and enabling clinical translation of TEP diagnostics at scale.^62^, ^87^, ^93^

### 5.2 Analytical harmonization and bioinformatic rigor

Platform heterogeneity and, critically, bioinformatic pipelines (normalization and statistics) remain major sources of variability.^21^ Establishing standardized, transparent, and validated bioinformatic workflows is imperative.^98^ This includes selecting validated reference genes or synthetic spike‐in controls for normalization, defining acceptable molecular quality metrics, and deploying robust methodologies for batch correction (e.g., ComBat‐Seq). Crucially, analytical protocols must pre‐specify biological covariates to prevent over‐correction and incorporate quantitative “do‐no‐harm” checks to confirm that technical noise is suppressed without eroding the underlying tumor‐specific signal.^99^

To operationalize these principles, we propose the following minimum ML reporting standards: (1) data independence: training and validation sets must be strictly separated (e.g., by hospital site or collection time) to preclude data leakage.^100^, ^101^, ^102^ (2) Nested feature selection: feature selection and hyperparameter tuning must occur *within* cross‐validation loops, never on the full dataset prior to splitting.^101^, ^102^ (3) Sample size and power: to mitigate overfitting in high‐dimensional platelet transcriptomics, studies should aim for an events‐per‐variable (EPV) ratio of >10–20 or employ strict dimensionality reduction.^103^ Crucially, for clinical validation, sample sizes must be determined by power analysis targeting precision for positive/negative predictive values (PPV/NPV) at the intended prevalence, rather than relying solely on AUC.^104^

### 5.3 Deciphering the signal: The challenge of confounding activation

A central challenge in translating TEPs to the clinic is the biological imperative to distinguish the true, tumor‐specific “education” signature from the confounding “noise” of generalized platelet activation.^105^ Even in health, platelet transcriptomes shift with age, diet, exercise, and subclinical inflammation; comorbidities and medications add further variability, fueling concerns that specificity ceilings may limit asymptomatic screening.^106^, ^107^ This baseline plasticity, compounded by comorbidities and medications, fuels skepticism that specificity ceilings may preclude asymptomatic screening.^105^

Addressing signal‐to‐noise is not merely theoretical but quantitatively critical. In the pan‐cancer validation by In 't Veld et al., specificity dropped from 99% (95% CI 95–100) in asymptomatic controls to 78% (95% CI 73–82) in symptomatic controls with non‐malignant conditions, illustrating the substantial false‐positive burden imposed by benign inflammation.^2^ Furthermore, drug‐induced transcriptomic shifts must be accounted for; Myers et al. demonstrated that antiplatelet agents, such as aspirin and ticagrelor, significantly alter platelet gene expression profiles—specifically upregulating mitochondrial and cytoskeletal pathways—which can confound cancer‐specific signatures if not bioinformatically isolated.^108^, ^109^ Therefore, distinguishing true “education” from these pharmacological and inflammatory “activations” is a prerequisite for clinical utility.

Our roadmap assumes that rigorous standardization and multi‐omic deconvolution can elevate the tumor‐specific signal above this background.^32^ Acute inflammatory events (e.g., infections, surgery, thrombosis) can dramatically alter platelet molecular profiles through non‐specific activation and may overlap with education signals, risking false positives.^110^, ^111^ Recent evidence reinforces that systemic inflammatory states—often quantified by metrics like the systemic immune‐inflammation index (SII) or platelet‐to‐lymphocyte ratio (PLR)—strongly influence cancer prognosis and reflect a host milieu that may distort platelet RNA profiles independent of specific tumor education.^112^, ^113^ Furthermore, therapeutic interventions themselves introduce variability. A critical, often overlooked factor is antiplatelet therapy (e.g., aspirin, P2Y12 inhibitors), which fundamentally alters reactivity and could mask or mimic TEP features. Similarly, immunotherapy has been shown to drastically modulate platelet counts and function, potentially altering the “educated” phenotype mid‐treatment.^114^ Addressing signal‐to‐noise requires: (i) trial designs that pre‐specify, stratify for, and document these inflammatory and iatrogenic conditions, and (ii) analytical strategies (below) that bioinformatically isolate education from activation.^32^

### 5.4 Designing definitive clinical trials

Retrospective case–control studies are insufficient. Definitive evidence must come from large, prospective trials demonstrating clinical utility—that TEP‐guided care improves outcomes or materially optimizes decision‐making.^37^, ^115^

To rigorously operationalize the management of potential confounders, we recommend a “Stratify and Adjust” trial design rather than broad exclusion criteria, which would limit real‐world generalizability.^116^, ^117^ We propose a specific framework for prospective implementation: (1) exclusion of acute factors: patients with acute inflammatory events (e.g., active sepsis, major surgery within 4 weeks) should be temporarily excluded, as acute‐phase reactant storms likely mask tumor‐specific signals. (2) Stratification of chronic factors: patients with chronic comorbidities (e.g., autoimmune disease, stable cardiovascular disease) or those on long‐term antiplatelet therapy should be included but stratified a priori. Statistical models must be powered to treat these factors as covariates to define distinct background noise profiles. (3) Mandatory metadata: to enable this adjustment, future trials must capture a “Minimum Clinical Data Set” for every sample: precise medication history (specifically dose and duration of aspirin and P2Y12 inhibitors), comorbidity burden (e.g., Charlson Comorbidity Index),^117^, ^118^ and systemic inflammatory markers at the time of draw (CRP, neutrophil‐to‐lymphocyte ratio).^119^, ^120^

Trials should target pressing unmet needs:

- MCED in asymptomatic populations. Large cohort studies (*n* > 10,000) to evaluate performance in intended‐use screening groups.^46^, ^121^ Endpoints should include positive and negative predictive value (PPV/NPV), maintenance of specificity ≥99%, and optimized diagnostic work‐up after a positive test.^45^
- Risk stratification of indeterminate pulmonary nodules (IPNs): a solution to the LDCT false‐positive burden. While broad MCED screening is the ultimate ambition, risk stratification of LDCT‐detected nodules offers a pragmatic path to near‐term utility and regulatory approval—leveraging an existing screening framework, meeting a clear clinical need, and enabling a definitive endpoint: reducing unnecessary invasive procedures without compromising cancer detection. Landmark trials show >95% of positive first‐round LDCT screens are not cancer, yielding high volumes of IPNs and downstream biopsies/resections with non‐trivial morbidity.^72^, ^122^

We propose a randomized trial of TEP + LDCT versus LDCT alone for managing IPNs. Based on a baseline unnecessary invasive procedure rate of ~25% for intermediate‐risk nodules (derived from NLST/NELSON data),^123^ a sample size of ~1800 patients is estimated to detect a 50% relative reduction with 90% power.

The assay should employ a dual‐threshold strategy: a “High‐Sensitivity” mode (HighSens, NPV >98%) to safely assign patients to surveillance de‐escalation, and a “High‐Specificity” mode to justify immediate intervention.^124^ A positive TEP result triggers guideline‐concordant escalation strictly targeted to the index nodule (e.g., PET‐CT, bronchoscopy), avoiding broad systemic work‐ups. The primary endpoint is reduction in unnecessary invasive procedures in the TEP + LDCT arm without delaying or reducing timely cancer diagnosis; co‐primary or key secondary endpoints should include time‐to‐diagnosis and stage shift among true cancers.

### 5.5 Beyond transcriptomics: A multi‐omics imperative for signal clarification

TEP transcriptomics has been foundational,^1^ but the recent description of pDNA in small mechanistic studies argues for a multi‐omics view and highlights an emerging analyte whose diagnostic utility is not yet defined.^3^ A single analyte is limiting. Integrating the transcriptome (RNA), proteome, and genome/epigenome (pDNA) is essential to clarify signal origin.^86^ Specifically, we propose a synergistic strategy combining TEP transcriptomics with plasma DNA methylation profiling. While TEP RNA signatures function as sensitive, dynamic sensors of malignancy and systemic inflammatory response, cfDNA methylation patterns offer highly specific, stable “epigenetic fingerprints” for tissue‐of‐origin localization. Co‐analyzing these orthogonal layers can mitigate the sensitivity gap of ctDNA in low‐shedding early‐stage cancers while reinforcing diagnostic specificity. For instance, in glioma progression, specific platelet RNA cargo has been shown to mirror key oncogenic drivers, including Wnt/β‐catenin signaling and specific RNA modifications (e.g., m6A regulators), offering a non‐invasive window into the tumor's molecular evolution.^125^, ^126^ Tumor‐induced inflammation can elevate both oncogenic and generalized inflammatory transcripts; multi‐omics resolves ambiguity by requiring concordance across orthogonal layers.^63^, ^127^ Example: a transcriptomic signature that includes both an oncogene (education) and a general inflammatory marker (activation) is ambiguous; if concurrent proteomics confirms the oncogenic protein while systemic inflammatory proteins are low, attribution favors tumor‐specific education.

In practical terms, a minimal multi‐omics panel could couple (i) a platelet‐RNA module capturing tumor‐associated transcripts and tissue‐of‐origin/splicing signatures alongside a predefined activation/inflammation module,^1^, ^32^ (ii) a focused proteomic panel quantifying canonical activation markers (e.g., PF4/TSP‐1‐like programs) versus selected tumor‐related proteins such as pPD‐L1,^82^, ^128^ and (iii) a targeted pDNA panel querying a small set of recurrent driver mutations or copy‐number events aligned with the intended cancer spectrum. Concordance rules can then be specified a priori: “education‐dominant” samples require elevation of the tumor RNA module together with a matching pDNA or tumor‐protein signal in the context of low activation scores; “activation‐dominant” samples show high activation/inflammation modules without tumor‐specific RNA/pDNA/protein signals and are interpreted as negative; mixed or discordant profiles are classified as indeterminate and trigger repeat sampling or orthogonal testing rather than immediate escalation. Such explicit, rule‐based integration makes the distinction between activation and education operational for both trial design and eventual clinical reporting. Ultimately, this comprehensive profiling lays the foundation for “theranostic” convergence. Emerging bioengineering approaches demonstrate that platelet‐informed platforms can facilitate responsive therapeutic delivery, paving the way for next‐generation strategies where engineered carriers are directed by the specific tumor‐platelet interactions identified via liquid biopsy.^129^, ^130^

### 5.6 Implementation, regulatory, and ethical landscape

Even with strong validation, implementation requires clear clinical guidelines and compelling health‐economic data to secure regulatory approval (FDA, EMA) and payer coverage.^131^ A positive MCED TEP result may create a “cancer of unknown primary,” raising ethical challenges in communicating likely malignancy without localization and risking low‐yield work‐ups; predefined diagnostic pathways and careful patient‐communication strategies are essential.^32^, ^45^, ^132^

### 5.7 Future perspectives and key unanswered questions

Successful implementation ultimately depends on deeper, mechanistic understanding.^1^, ^133^ We consolidate critical open questions in Box 2 to guide basic and translational work.

BOX 2 **Key Unanswered Questions in TEP Biology.**

While the diagnostic potential of TEPs is evident, fundamental gaps in our biological understanding remain. Answering the following questions is critical for moving the field from phenomenological observation to mechanistic validation, which is essential for developing next‐generation, robust TEP‐based diagnostics.

- The locus of education: central versus peripheral imprinting: is imprinting predominantly peripheral (uptake by circulating platelets; dynamic, response‐sensitive) or central (altered megakaryopoiesis; stable, time‐integrated)? Clinical implications differ for diagnosis versus response monitoring.^105^, ^134^
- Mechanisms of education: which tumor‐derived factors (extracellular‐vesicle [EV] cargo, soluble proteins, metabolites) drive education, how do they vary by cancer type, and how do they evolve from premalignancy to advanced disease?^135^, ^136^
- Heterogeneity of the signal: how do inter‐patient and intra‐tumor heterogeneity shape TEP profiles, and can robust subtype‐specific signatures be defined?^2^
- Platelet‐level resolution: can single‐platelet sequencing identify a highly “educated” subpopulation with superior diagnostic specificity?^89^
- Impact of host factors: how do comorbidities (e.g., chronic inflammation, cardiovascular disease), medications (e.g., antiplatelets, statins), and hematopoietic context (e.g., clonal hematopoiesis) modulate or confound TEP signatures—and can these effects be deconvoluted?^2^, ^108^, ^109^
- Mechanisms of DNA sequestration: what pathways govern uptake of membrane‐free DNA versus EV‐encapsulated DNA by platelets?
- The fate of internalized DNA: is sequestered DNA inertly stored or processed prior to release upon activation? Could platelets mediate horizontal gene transfer?
- The RNA–DNA interplay: within single platelets, do specific tumor‐derived RNAs correlate with ctDNA sequestration, implying coordinated education?

## 6 CONCLUSION

**FIGURE 4:**
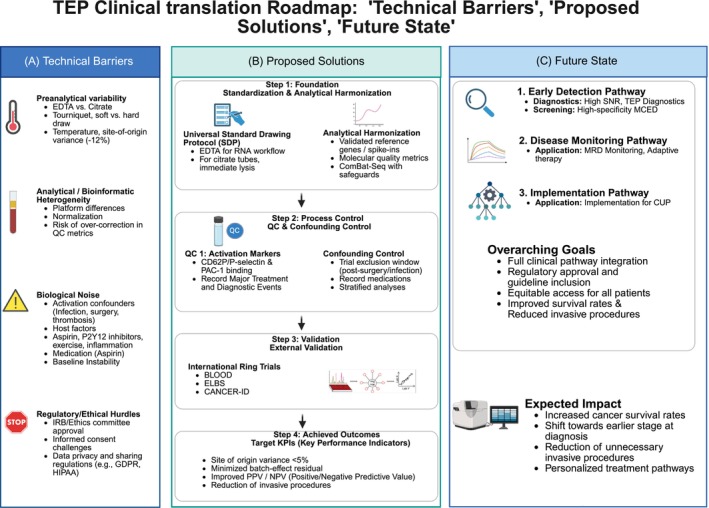
TEP clinical translation roadmap: “Technical Barriers,” “Proposed Solutions,” and “Future State.” (A) Technical barriers. This panel categorizes the primary obstacles hindering the clinical adoption of TEP assays into four key domains: preanalytical variability (e.g., anticoagulant choice, storage), analytical/bioinformatic heterogeneity (e.g., platform differences, normalization), biological noise (e.g., confounding factors like inflammation or medication), and regulatory/ethical hurdles regarding data privacy and approvals. (B) Proposed solutions. A four‐step strategic framework is outlined to overcome these barriers. Step 1 (foundation) establishes data consistency through universal standard drawing protocols and analytical harmonization. Step 2 (process control) ensures reliability via rigorous quality control of activation markers and management of confounding factors. Step 3 (validation) demonstrates robustness through external validation measures such as international ring trials. Step 4 (achieved outcomes) aims to meet target key performance indicators (KPIs), such as minimized site‐of‐origin variance and improved predictive values. (C) Future state. The final panel illustrates the vision following successful implementation. It delineates three clinical pathways: early detection (screening/diagnostics), disease monitoring (e.g., MRD assessment), and implementation for specific indications (e.g., CUP). Furthermore, it summarizes the overarching goals, such as full clinical integration and regulatory approval, alongside the expected impact on patient care, including increased survival rates and reduced unnecessary invasive procedures (Created with BioRender.com). CANCER‐ID, cancer‐ID liquid biopsy consortium; CUP, cancer of unknown primary; EDTA, ethylenediaminetetraacetic acid; ELN, electronic laboratory notebooks; GDPR, general data protection regulation; HIPAA, Health Insurance Portability and Accountability Act; KPI, key performance indicator; MCED, multi‐cancer early detection; MRD, minimal residual disease; NPV, negative predictive value; PPV, positive predictive value; QC, quality control; SDP, standard drawing protocol; SNR, signal‐to‐noise ratio; TEP, tumor‐educated platelet.

TEPs represent a distinct and highly informative domain within the liquid biopsy landscape, capturing a dynamic, integrated signature of malignancy across multiple omic layers. Foundational research has unequivocally demonstrated the capacity of TEP molecular profiles—primarily transcriptomic—to detect the presence of cancer and localize the tissue of origin in controlled research settings. The field now stands at a critical inflection point: the translation of this powerful proof‐of‐concept into a clinically robust diagnostic modality (Figure 4).

Achieving this translation demands a rigorous, concerted, multi‐disciplinary effort focused on standardization and validation. The immediate imperatives are the establishment of universal SOPs—including stringent QC metrics for platelet purity and activation—and the execution of large‐scale prospective trials designed to demonstrate clinical utility in real‐world populations. Furthermore, the ultimate potential of TEPs will likely be realized through integration. Fusing TEP data with orthogonal analytes, such as ctDNA, and employing comprehensive multi‐omics approaches (transcriptomics, proteomics, and lipidomics) will be essential for maximizing diagnostic sensitivity and specificity.

While the path to routine clinical adoption is challenging, TEPs are poised to become a vital component of the liquid biopsy armamentarium. Sustained scientific rigor, adherence to standardized protocols, and collaborative validation efforts will ensure that the compelling initial data culminates in tangible clinical tools that improve patient outcomes through earlier, more precise cancer diagnosis and management. Furthermore, a deeper understanding of the platelet “education” mechanism—including the emerging concept that platelets can capture both the tumor's expressed messages (RNA) and fragments of its genomic blueprint (pDNA)—not only perfects our diagnostic tools but may also inspire novel anti‐metastatic therapeutic strategies targeting these platelet‐tumor interactions in the future.

## AUTHOR CONTRIBUTIONS

**Whi‐An Kwon:** Conceptualization; writing – original draft; writing – review and editing; visualization. **Min‐Kyung Lee:** Writing – review and editing; supervision. **Eunyong Ahn:** Writing – review and editing; validation; methodology. **Heeyeon Kim:** Writing – review and editing; validation; investigation. **Yong Sang Song:** Supervision; methodology; resources. **Taejin Ahn:** Conceptualization; supervision; resources.

## CONFLICT OF INTEREST STATEMENT

Authors Eunyong Ahn, Heeyeon Kim, and corresponding author Taejin Ahn are affiliated with the Research Group, Foretell My Health. The remaining authors declare no conflicts of interest.
